# Cellular plasticity in the breast cancer ecosystem

**DOI:** 10.48101/ujms.v129.10629

**Published:** 2024-04-02

**Authors:** Kristian Pietras, Jonas Sjölund

**Affiliations:** Department of Laboratory Medicine, Division of Translational Cancer Research, Lund University Cancer Centre, Medicon Village, Lund University, Lund, Sweden

**Keywords:** Tumor microenvironment, tumor-initiating cells, cancer-associated fibroblasts, macrophages

## Abstract

The complex interplay between genetically diverse tumor cells and their microenvironment significantly influences cancer progression and therapeutic responses. This review highlights recent findings on cellular plasticity and heterogeneity within the breast cancer ecosystem, focusing on the roles of cancer-associated fibroblasts (CAFs) and tumor-associated macrophages (TAMs). We discuss evidence suggesting that breast cancer cells exhibit phenotypic plasticity driven by both intrinsic genetic factors and external microenvironmental cues, impacting treatment responses and disease recurrence. Moreover, single-cell RNA sequencing studies reveal diverse subtypes of CAFs and TAMs, each with distinct functional gene expression programs and spatial organization within the tumor microenvironment. Understanding the hierarchical relationships and niche cues governing cellular phenotypes offers new opportunities for targeted therapeutic interventions. By elucidating the organizational principles of the tumor ecosystem, future therapies may target phenotypic states or entire cellular niches, advancing precision medicine approaches in breast cancer treatment.

## Introduction

Clinically manifested malignant disease results from an interplay between genetically altered tumor cells with their appropriated micro- and macro-environment (1, 2). The cancer ecosystem is shaped through reciprocal paracrine signaling events that alleviate barriers to malignant transformation. Efforts to carefully map tumor-supportive and -restrictive functions of the diverse constituent cell types within the cancer environment have resulted in the development of targeted treatment approaches, including drugs acting on endothelial cells, immune cells, and mesenchymal components (3). Yet, only a minute fraction of cellular interactions has been therapeutically exploited, and the full potential of targeting microenvironmental support or reinforcing stromal resistance can only be realized by delineating the organizational principles of the tumor organ.

One of the greatest challenges to achieving cures with current treatment modalities for malignant disease is the tremendous genetic heterogeneity observed in cancer cells, since cellular diversity and plasticity provide the basis for therapeutic resistance, manifested both as primary and as acquired refractoriness to treatment. The most striking example of this is provided by demonstration of tumor cells with stem-like properties that harbor intrinsic drug resistance (4). However, it is still unclear whether cancer cells are hierarchically organized similar to embryonic or somatic stem cell phylogenies, or whether a more stochastic model applies. Importantly, phenotypic heterogeneity is not restricted to the malignant compartment but prevalent within the genetically stable tumor microenvironment (5, 6). New and powerful technologies allowing for the enumeration of cellular phenotypes with single-cell resolution have invigorated exploration into transcriptionally discrete subsets of microenvironmental cell types, most notably for cancer-associated fibroblasts (CAFs) and myeloid cells. Again, lineage relationships and complete taxonomies of subsets of these broadly defined cell types are yet to be allocated.

Here, using breast cancer as a prototypical solid tumor, we review literature on cellular plasticity within different compartments of the cancer ecosystem. Moreover, we propose that phenotypic diversity derives from environmental cues within cellular microniches in which paracrine signaling networks are established.

## Malignant cell plasticity

Breast cancers are manifested as several different subtypes of disease. Based on biomarker expression, pathological analyses define malignancies as positive for estrogen receptor (ER), progesterone receptor (PR), and HER2, or as negative for all three markers (triple-negative breast cancer, TNBC). Molecular subtyping based on derivation of the PAM50 classifier (7) by RNA sequencing largely confirms the pathological subtypes as transcriptionally separate entities, defining tumors as luminal A (ER^+^, PR^+^, and HER2^-^), luminal B (ER^+^, PR^+/-^, HER2^-^; Ki67^high^), HER2-enriched (HER2^+^, ER^+/-^, and PR^+/-^), or basal-like (overlapping with the TNBC subtype to ~70%). The molecular characteristics of these subtypes direct treatment modalities, as patients with ER^+^ tumors receive various forms of endocrine treatment, and patients with HER2^+^ tumors benefit from targeted therapies toward the HER2 receptor (8). TNBCs have the highest rate of recurrence and the worst 5-year overall survival. However, in contrast to TNBCs that rarely recur after 5 years, luminal breast cancers may reemerge up to 20 years after diagnosis, indicating differences in their ability to induce or uphold dormancy (9).

The discordance between the molecular subtype of primary breast cancers and their respective recurrent tumors indicates that there is some degree of phenotypic plasticity that may be exploited for therapeutic purposes (10). Whereas the most common event is that recurrent luminal tumors reemerge as TNBCs, the converse is also observed (10). Such plasticity may be explained by recent evidence that luminal and *BRCA*-mutated basal-like breast tumors share a common origin from luminal progenitors (11, 12). Indeed, the TNBC phenotype appears in part to be encoded epigenetically, as treatment of cell cultures with histone deacetylase inhibitors will efficiently switch cells from an ER^-^ to an ER^+^ state by resetting any previous differentiation marks (13, 14). Additionally, a signaling pathway emanating from polo-like kinase (PLK) 1 was recently demonstrated to also induce ER-α expression in TNBC cells (15). Intriguingly, PLK1 phosphorylation of the transcriptional repressors SUZ12 and ZNF198 enhances their proteasomal degradation, thereby causing widespread dysregulation of histone modifications (16). The specification of molecular subtypes of breast cancers is, however, not only intrinsically regulated but also under microenvironmental control. We have demonstrated that paracrine stimulation of CAFs by platelet-derived growth factor (PDGF)-CC supplied by TNBC cells upregulate a cocktail of secreted mediators that suppress hormone receptors and their transcriptional mediators (17). Accordingly, the inhibition of PDGF-CC converts TNBC into ER-α^+^ cancer *in vivo*, accompanied by an induced sensitivity toward endocrine therapy. The luminal gene expression program is, in this context, induced genome-wide, as demonstrated by integrated ChIP-seq and RNA-seq experiments. Conversely, hormone signaling in luminal breast cancer cells is selectively repressed by the juxtaposition of CAFs, both *in vitro* and *in vivo* (18, 19). The functional importance of cellular plasticity is further demonstrated by the fact that CAFs maintain phenotypes in breast cancer cells reminiscent of stem-like properties, such as drug resistance and invasion, while quelling more differentiated properties, such as hormone dependence (18). Recent studies of myeloid cells demonstrate also their ability to regulate the plasticity of breast cancer cells in order to support a stem-like character (20). Here, cancer cell-derived cytokines educate myeloid cells to reciprocate by secreting oncostatin M and IL-6, which, in turn, induces cancer-stem cell (CSC) properties in the malignant cells.

Taken together, breast cancer cells are highly plastic, and their phenotypic identity is governed by both cell-autonomous (genetic) and environmental factors.

## Breast CAF subtype heterogeneity

It is well-established that the tumor microenvironment is instrumental during tumor initiation, manifestation, dissemination, and in shaping the response to therapy. Early studies of CAF heterogeneity indicated the coexistence of at least two subsets with distinct spatial organization, that is, myofibroblast (my)CAFs that reside in juxtaposition to the malignant epithelium and inflammatory (i)CAFs that are positioned deeper within the stroma (21). Recent analyses at single-cell resolution confirm a high degree of heterogeneity among stromal cell types and, in particular, among CAFs. Utilizing single-cell RNA-seq of advanced stage tumors from the prototypical MMTV-PyMT genetically engineered mouse model of breast cancer, we designated three subtypes of CAFs that were distinguished by their origin and accompanying functional gene expression programs (22). The most prevalent breast CAF in overtly invasive carcinomas originates from a peri-vascular location, harbors a gene expression program related to vascular function, and was consequently termed vascular CAF (vCAF). The abundance of vCAFs is consistent with this subtype being the only one with an associated subcluster of cycling CAFs (cCAF). With tumor progression, vCAFs detach from the vasculature and populate the tumor stroma predominantly within the tumor core. The second most prevalent breast CAF derives from the resident fibroblasts supporting the normal mammary gland. This subtype was termed matrix CAF (mCAF), owing to their prolific production of extracellular matrix components, including the prototypical collagen I. Finally, a rare subtype of CAFs was identified represented by malignant cells that had undergone an epithelial-to-mesenchymal transition (EMT) and manifests a developmental gene expression profile, hence their denomination as developmental CAF (dCAF). The coexistence of several subsets of CAFs in mammary carcinomas was corroborated in tumors derived from the TNBC cell line 4T1 (23). Transcriptionally distinct CAFs were, in this case, delineated by the expression of podoplanin (pCAFs) or S100A4 (FSP1; sCAFs). Intriguingly, a higher ratio between sCAFs and pCAFs correlated with a prolonged recurrence-free survival. Unexpectedly, a lower abundance of pCAFs was also observed in TNBCs with *BRCA1* mutations, suggesting that the malignant cell genotype in part directs the organizational principles of the stromal compartment.

An even higher complexity of breast CAF subsets was delineated in human breast cancers (24, 25). In particular, the CAF-S1 subset of these studies was further dissected into eight distinct clusters. The functional units were distinguished by their abilities to regulate the immune system, in particular T cells, and to remodel the extra-cellular matrix. The most comprehensive study of the human breast tumor microenvironment to date largely corroborates earlier findings (26), defining two types of CAFs, each with several substrates, broadly corresponding to myCAFs and iCAFs. In addition, two related perivascular-like (PVL) mesenchymal cells designated immature (with a pericyte-like gene expression profile) and differentiated (with a vascular smooth muscle cell-like gene expression profile) were identified, reminiscent of the vCAF subset identified by us.

Thus, recent technological advances have propelled our knowledge about the diverse functional gene expression programs harbored by distinct CAF subtypes. However, the organizational principles in relation to other microenvironmental elements are still unknown. Also, the underlying tenets behind the division of CAF subsets are not fully understood, as the lineage relationship between different CAF clusters, if any, has not been fully established.

## Macrophage diversity

Myeloid cells come across as cells with a remarkable inherent plasticity. Several broadly defined cell types, such as monocytes, macrophages, dendritic cells, and myeloid-derived suppressor cells (MDSCs), perform distinct functions, for example, when it comes to immunosuppression, yet share a single common progenitor cell, as well as similar basic characteristics. The studies of the cellular landscape of human breast cancer by Wu et al. (26) defined 13 clusters of myeloid cells (excluding granulocytes that were not captured during the isolation procedure). Apart from monocytes and dendritic cells, macrophages constituted almost half of the myeloid clusters with six distinct transcriptomic profiles, including one cluster of proliferating cells. The previously suggested dichotomization of macrophages into ‘M1’ and ‘M2’ activation states based on their phenotypic similarity with classically activated macrophages versus alternatively activated macrophages is represented by two ‘M1’-like and one ‘M2’-like clusters. Interestingly, the ‘M2’-like cells also express the highest levels of a gene signature for recruited tumor-associated macrophages (TAMs). In support of this model, a study by Azizi et al. similarly describes three transcriptionally distinct subsets of macrophages (27). However, both ‘M1’ and ‘M2’ characteristics appear to be shared among these subtypes, demonstrating how dimensionality reduction by different methodologies may visualize the data from discrete perspectives. Additionally, two subsets of lipid-associated macrophages (LAMs) were described (26). The function of the LAM subtypes is yet to be uncovered, but according to their transcriptional program, they express a wide variety of immunoregulatory cytokines and receptors, making them interesting subjects for biomarker and therapeutic target explorations.

In summary, single-cell RNA-seq studies confirm the emerging picture that there is more to TAM diversity than the previously described ‘M1’ and ‘M2’ phenotypic states. Yet, how macrophage diversity is shaped by the organizational principles of the tumor ecosystem, and reciprocally shapes cellular and molecular microniches, remains to be determined.

## Perspective

Recent technological leaps have enabled a more detailed view of heterogeneity within the tumor ecosystem. Evidently, the basis for such heterogeneity is not the same for all cellular compartments. Integration of single-cell RNA-seq data from cohorts of cancer patients illustrates that malignant cell clusters maintain the original patient identity, indicating that genomic parameters override environmental cues in determining heterogenic patterns (26). Nevertheless, despite the fact that each patient harbors malignant cells with a dominant molecular subtype, all tumors exhibit significant heterogeneity when predicting the PAM50-based subtype of cancer cells on an individual basis, still suggesting an influence from the surrounding niche. In contrast to malignant cells, microenvironmental cell types cluster in a patient-agnostic manner in high-dimensional transcriptional analyses. This may be explained by the fact that non-epithelial cell types are products of their environment and have an acute sensing ability. In particular, CAFs and macrophages are specialized in perceiving environmental cues and reacting to them, hence their extreme diversification with regard to cellular states.

**Figure 1:**
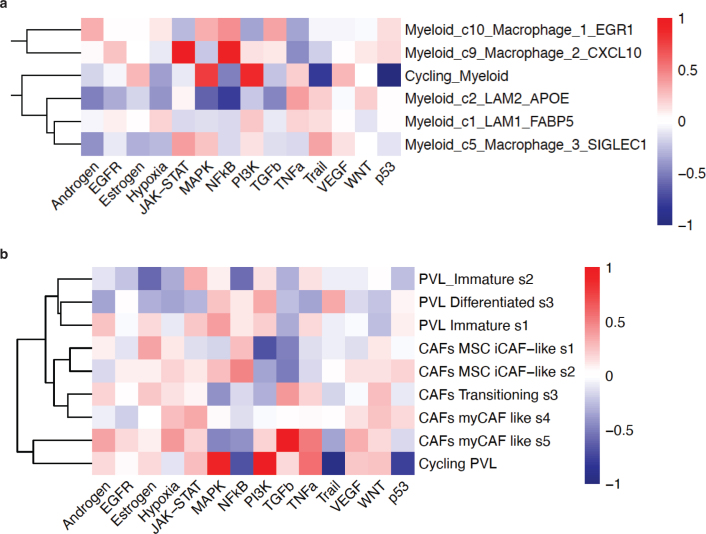
Microenvironmental heterogeneity revealed by pathway activity analysis. Pathway activities were estimated using the PROGENy tool (30) on the dataset by Wu et al. (26). Macrophage (a) and cancer-associated fibroblast (CAF)/perivascular-like fibroblast (PVL) (b) subtypes are characterized by distinct patterns of pathway activation. Notably, subtypes of each cell type exhibit similar activation patterns, suggestive of spatial co-localization in discrete niches.

Cellular heterogeneity may result from fixed hierarchies; such stable phylogenies are prevalent during embryogenesis or from somatic tissue stem cells. In the context of malignant disease, hierarchical relationships have been suggested from drug-resistant CSCs that give rise to amplifying bulk cells (28). However, recently, it has been necessary to modify the CSC hypothesis to take the microenvironment into account (29). Evidently, the cellular hierarchies of CSCs are more plastic than previously thought. In the most extreme case, plasticity would be boundless, and even cellular identity would be governed in full by the environmental context. We will exemplify this train-of-thought with microenvironmental cell types at different ends of the plasticity spectrum. On the one hand, T cell identities result from a fixed phylogeny emanating from a lymphoid-committed progenitor cell, with fine-tuning of the phenotype by environmental cytokine cues. On the other hand, macrophages and CAFs appear to derive from a flat hierarchy that is significantly influenced by their local milieu. In support of the impact of the niche in shaping cellular subtypes, we have estimated signaling pathway activity in macrophages and CAFs from the study of human breast cancers by Wu et al. using the PROGENy tool (30). Strikingly, each subset of cells exhibits a distinctive pathway activation profile (Fig. 1A-B), reinforcing the extrinsic influence on diversification of the cellular subtypes. Thus, cells in Macrophage cluster 2 – CXCL10 have an extreme activation of both JAK-STAT and NF-κB signaling, whereas cells in Macrophage cluster 3 – SIGLEC1 are characterized by Trail activation (Fig. 1A). Similarly, cells in myCAF-like state 5 are distinguished by TGF-β pathway activity, in contrast to cells in iCAF-like state 2 that instead exhibit high activation of NF-κB signaling (Fig. 1B). Intriguingly, macrophage and CAF clusters that harbor transcriptional programs with similar activation profiles are evident. It would come as no surprise that cycling macrophages and cycling PVLs share a MAPK and PI3K activation profile (Fig. 1A-B). However, more unexpectedly, similarities between subsets of macrophages and CAFs/PVLs are readily evident. As an example, both cells in Macrophage cluster 2 – CXCL10 and cells in myCAF-like state 4 exhibit among the highest JAK-STAT and p53 pathway activities within each respective cell types (Fig. 1A-B). Similarly, both cells in Macrophage cluster 1 – EGR1 and cells in the myCAF-like state 5 have the highest activation of TGF-β and androgen signaling within their cell type (Fig. 1A-B). It is tempting to speculate that similar pathway activation profiles in different cell types result from sharing of the same cellular niche, thereby experiencing the same molecular cues. Indeed, cells in Macrophage cluster 2 – CXCL10 and in myCAF-like clusters exhibit the closest proximity to CD8^+^ T cells, according to spatial transcriptomics analyses (26), indicative of a tripartite cellular niche composed of distinct subsets of macrophages, CAFs, and T cells. In the most extreme iteration of this proposition, cellular origin is rendered irrelevant, and the organizational principles of the tumor ecosystem dictate cellular phenotypes by fluently inducing trans-differentiation. Indeed, transition of monocyte/macrophages into myofibroblasts has been reported in a few studies (31, 32). However, most likely, tumors are built by a middle ground between stable hierarchies and absolute plasticity.

Taken together, it is increasingly evident that to understand cellular heterogeneity within the tumor ecosystem, we need to take both the cellular origin and the environmental context into account, since they are concertedly determining the phenotype. The full understanding of hierarchical relationships and niche cues offers the prospect of developing future therapies against malignant disease by targeting cellular niches or phenotypic states of the tumor ecosystem, rather than single pathways.
